# Transition to Adulthood through Coaching and Empowerment in Rheumatology (TRACER): A feasibility study protocol

**DOI:** 10.1371/journal.pone.0295174

**Published:** 2024-08-26

**Authors:** Emma Reesor, Dorota Borovsky, Julie Herrington, Pamela Jarvis, Megan Clarke, Roberta Berard, Karen Beattie, Michelle Batthish

**Affiliations:** 1 McMaster University, Hamilton, Ontario, Canada; 2 McMaster Children’s Hospital, Hamilton Health Sciences, Hamilton, Ontario, Canada; 3 Division of Rheumatology, Department of Pediatrics, University of Western Ontario, London, Ontario, Canada; 4 Division of Rheumatology, Department of Pediatrics, McMaster University, Hamilton, Ontario, Canada; PLOS, UNITED KINGDOM

## Abstract

The transition from pediatric to adult care for patients with chronic disease is a vulnerable period, with risks of disengagement from care and subsequent complications of inadequately managed disease. This period comes at a time when there are many other transitions occurring in the young person’s life, including changes to vocation, social supports, and to their physiology. The aim of the TRACER study is to assess the feasibility of conducting a multi-center, randomized-controlled trial of a virtual Transition Coach Intervention in youth transferring from pediatric to adult rheumatology care. Patients are being recruited at their last pediatric rheumatology visit from McMaster Children’s Hospital and Children’s Hospital, London Health Sciences Centre in Ontario, Canada. Participants are then randomized to standard of care or to eight transition coaching sessions, covering topics around health management, future planning, and self-advocacy. The primary outcomes of the study are to demonstrate protocol feasibility, including optimal recruitment and consent rates, ≥ 90% coaching session completion, and complete data collection with ≤ 5% missing data. Baseline demographics, transition readiness, global functional assessment, disease activity, and self-efficacy will be collected to characterize the study population. Recruitment has begun and is estimated to last 19 months. This study will inform the design of a robust, multi-centered, randomized-controlled study to investigate the impact of a virtual transition coaching program in supporting the physical, mental, and social well-being of youth with rheumatic disease transitioning into adult care.

**Clinical trial registration:** ClinicalTrials.Gov protocol ID: 14499

## Introduction

Health Care Transition (HCT) is a purposeful, planned process where youth ideally receive developmentally appropriate support while progressively assuming more responsibility for their health as they prepare to transfer from pediatric to adult care [1, 2]. A poorly managed transition can have adverse effects on the quality and experience of care, as well as contribute to poor disease outcomes including morbidity and even mortality [1, 3–7]. Unfortunately, youth frequently report a negative experience during transition and as many as 50% disengage entirely from care [4]. Whilst preparing for and adjusting to the seemingly abrupt changes in care brought on by the transfer to adult rheumatology, youth are also experiencing several critical life transitions, including changes to their physiology (i.e., hormonal changes), social supports, and educational/vocational pursuits. In adult clinics, patients are expected to be competent and mature enough to manage their medications and chronic disease independently at age 18; while we know that their executive functioning and reasoning will be continuing to develop into their mid-twenties.

There has been growing interest in transition care research, however the period from age 18–25 (also known as the third phase of transition), occurring after transfer to adult care, remains disproportionally under-studied [2, 8–15]. There is a strong need for high quality studies of evidence-based transition programs, across a range of chronic diseases [9, 16] to inform best practices, improve the experiences for patients, and reduce loss to follow-up. Generally, transition programs have been found to be beneficial [17, 18], however there is significant variability in what the programs entail and even what outcome measures should be used as a metric of success [19, 20]. Recently, published HCT guidelines recommend developing individualized plans for HCT, interdisciplinary care, and utilization of technology to improve healthcare visit attendance [10, 15, 21].

Healthcare coaching (HC) has been described as a strategy for supporting patients as they transfer to adult care [22]. It has been defined as “helping patients gain the knowledge, skills, tools and confidence to become active participants in their care so that they can reach their self-identified health goals” [23]. HC has been demonstrated to have positive effects on quality of life, self-management, patient activation, and self-efficacy in patients with early-stage kidney disease [24]. While HC has been described as a form of transition support in rheumatology care [25], there is great potential for it to support youth prior to and, perhaps more importantly, after they transition to adult care.

Locally, at McMaster’s Young Adult Rheumatology clinic in Ontario, Canada, an informal coaching program was started in March 2020 with an Advanced Clinician Practitioner in Arthritis Care (ACPAC) physiotherapist. ACPAC training is a postgraduate certificate program based out of the Faculty of Medicine Continuing Education Department at the University of Toronto for experienced allied health professionals preparing them for advanced practice roles in rheumatology [26]. This informal coaching program was developed in response to the COVID pandemic/lockdowns and entailed a meeting with patients one week prior to their adult rheumatology appointment and provided support, education, and coaching. As a result of this intervention, youth reported feeling more confident in their care, and anecdotally there was an improvement in appointment attendance.

The TRACER study protocol was designed to demonstrate the feasibility of a transition coaching intervention and inform the design of a future larger multi-centre trial. This study is expected to provide protocol validation and experience to facilitate the next study phase, which will be powered to test intervention effectiveness. By conducting the research in this manner, we aim to create high-quality evidence around a structured intervention supporting youth during the critical third phase of HCT.

## Materials and methods

### Aim, design and setting of the study

The study aims to assess the feasibility of conducting a multi-center, randomized-controlled trial to answer the following research question: “*To what extent does the implementation of a Youth Transition Roadmap (YTR) and virtual Transition Coach Intervention (TCI) over the YTR alone improve self-management skills in youth being transferred from pediatric to adult rheumatology care*?*”* This study is based out of two academic centres in Ontario, Canada: McMaster Children’s Hospital, Hamilton Health Sciences, and Children’s Hospital, London Health Sciences Centre, with associated community rheumatologist and a satellite site in Windsor, Ontario. Feasibility will be assessed using the following criteria: consent rate, proportional enrollment from the secondary site, attendance of coaching sessions, completion of 8-month outcome assessments, and proportion of missing data. Feasibility trial success will be measured against the standards defined in Table 1, based on the CONSORT criteria for progression [27].

**Table 1:** Primary study aims to demonstrate protocol feasibility.

Feasibility Aims
Consent ≥85% of patients approached
Enroll ≥30% from non-primary site (London, Ontario)
≥90% attendance of virtual sessions
Complete ≥90% of outcome assessments at 8-month follow-up
≤5% missing data

The secondary aim of the study is to explore clinical outcomes at baseline, 8- and 11-months to characterize our study population and understand variability in changes over time in self-efficacy that may be used to power a large, multicentre RCT.

### Inclusion and exclusion criteria and sample size

All 17- and 18-year-old patients with a pediatric-onset rheumatic disease attending their last scheduled pediatric rheumatology appointment prior to transferring to adult care will be assessed for eligibility by their pediatric rheumatologist. Participants will be eligible if they are able to communicate in English, have access to a device capable of videoconference or a phone, and are available over the subsequent 8 months.

The sample size for this study was based on the more conservative of the two primary feasibility outcomes: ≥90% of TCI sessions attended. Assuming this, a sample size of 97 will be required for estimating the expected proportion with 6% absolute precision and 90% confidence [28]. With an 85% consent rate [29] and a 90% study completion rate (133 x 85% x 90% = 102), the target of 97 participants completing the study will be met.

### Recruitment and randomization

Patients will be recruited at their last scheduled pediatric rheumatology visit before transferring to adult care. The treating pediatric rheumatologist will inform the patient about the study and document reasons for ineligibility, as applicable. Interested patients will be directed to a research assistant who will provide further information and obtain consent. Consenting participants will be assigned a study identification number and randomly allocated to the TCI or the control group using a site-stratified computer-generated block randomization list with a block size of 4 and a 1:1 allocation ratio. Given the nature of the intervention, no blinding will be done. The study key containing the participants’ names and study identification numbers will be saved on a university server in a password protected folder accessible to only the research assistant and one additional member of the research team.

### Intervention

All participants (TCI and control group) will receive a paper and electronic copy of the Youth Transition Roadmap (YTR) [30] that is usual care, which informs youth about differences between adult and pediatric care and discusses 5 domains of healthcare transition: Self-Advocacy, Medication Management, General Health and Safety, Lifestyle Issues and Future Planning related to education and vocation (link, S1 File).

Participants in the TCI group are scheduled for monthly virtual (videoconference or phone call, depending on participant preference) sessions with a transition coach, with the first session within 2 weeks of consent. These sessions are scheduled independently of clinic visits and occur monthly for 8 months; 6 sessions with an Advanced Clinician Practitioner in Arthritis Care (ACPAC) Physiotherapist and 2 sessions with a Social Worker. The ACPAC physiotherapist sessions will include discussions about transition, self-advocacy, general health and medications, lifestyle and behaviours, future planning, and cultural difference between pediatric and adult care. The social worker sessions will include a screen for anxiety and depression using the Patient Health Questionnaire 4 (PHQ4) [31], and information tailored to areas of need identified by the participant based on the list in Table 2.

**Table 2:** Description of session topics for the transition coaching intervention group.

ACPAC Physiotherapist Sessions	Social Worker Sessions
1. Pediatric to adult care 2. Self-advocacy 3. Medication management 4. General health 5. Lifestyle and behaviours 6. Future planning	1. Screening for anxiety and depression (PHQ4) 2. Depending on the participant’s self-identified areas of need, topics including: • Disease severity, daily impact, current treatment, and possible side effects • Biologic life transitions • Self-management skills • Pain management and coping strategies • Body image, confidence • Living situation, parental/sibling support • Ability to partake in social/recreational activities • Social relationships/networks • Sleep/motivation/energy • Anxiety, depression, anger, denial (grief, feelings associated with loss) • Lack of validation from others • Communicating effectively with medical team/self-advocacy • Coping/accommodations with school • Being different than others • Becoming aware of negative thought patterns and education surrounding learning to recognize depressive thought patterns and strategies • Uncertainty/fear about the future

All participants will be scheduled for their first adult rheumatology visit within 3–6 months of consent. The timing of subsequent clinical visits will be at the adult rheumatologist’s discretion, based on their clinical judgment and the participant’s needs. All participants will receive standard of care from their rheumatologists and there is no restriction on medications, interventions or participation in other clinical trials.

### Outcome measures

This study is a pilot study, which will be used to inform a multi-centre trial measuring the impact of transition coaching for patients transitioning from pediatric to adult rheumatology. This study’s primary outcomes are feasibility measures as outlined above.

For the secondary aim of the study, data will be collected to characterize the population. Demographic data will be collected by chart review, including age, sex, gender, disease, current medications, age at initial diagnosis, family history of rheumatic disease, comorbidities, planned vocation (employment, post-secondary education, other), and estimate of household income (using postal code).

**Fig 1:**
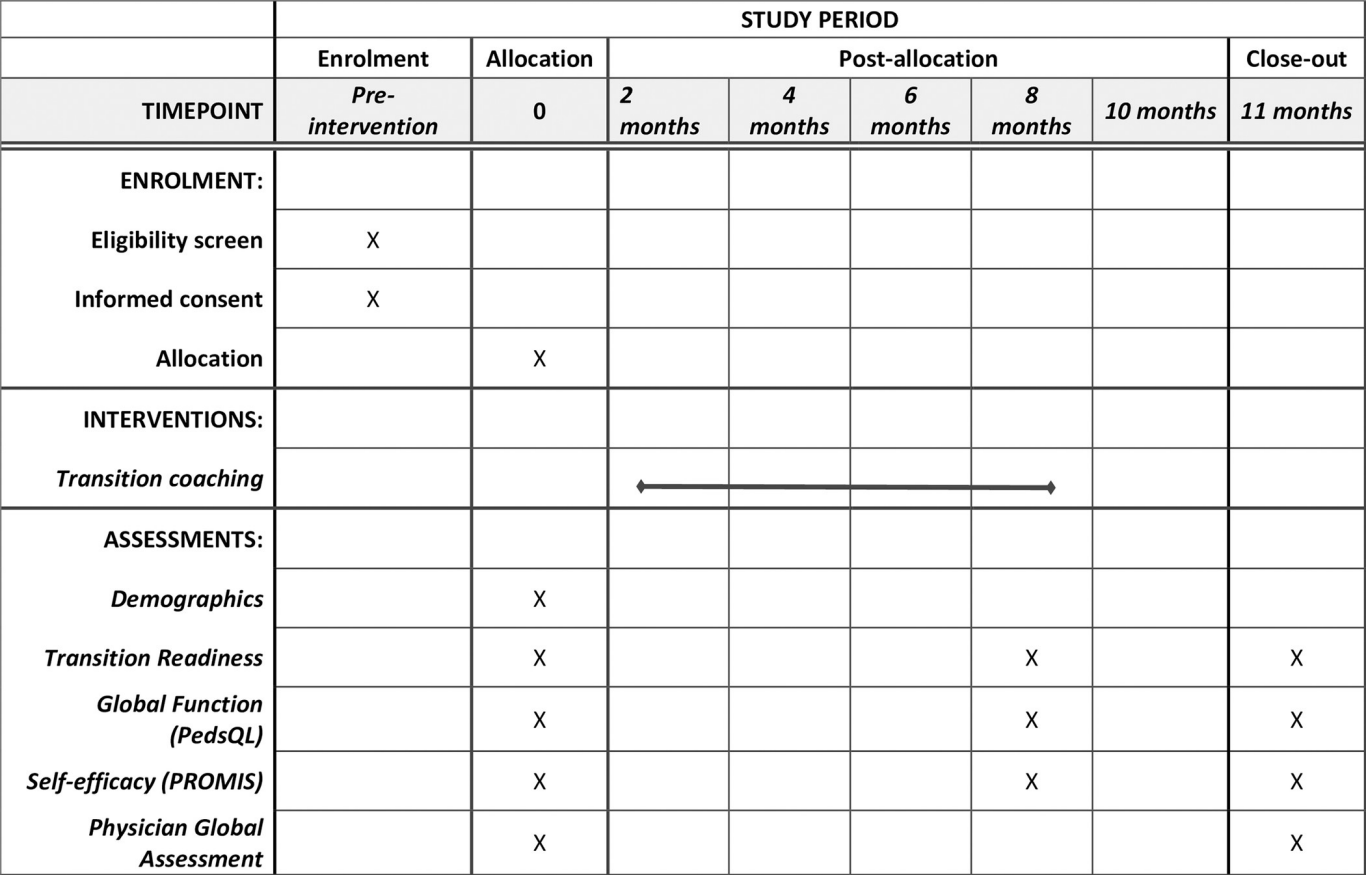
SPIRIT study timeline.

**Fig 2:**
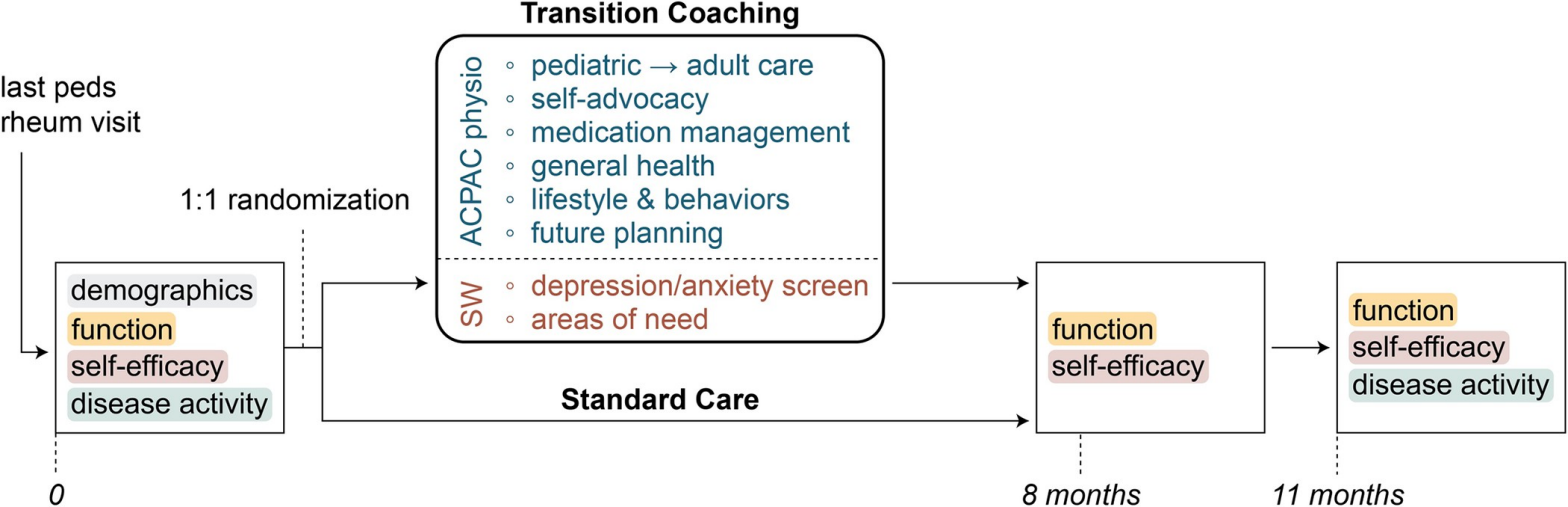
Graphical study timeline and overview.

Surveys will be administered to both groups at baseline, 8-months, and 11-months (3 months after the completion of the transition coaching intervention) to measure transition readiness, global function, and self-efficacy (Figs 1 and 2). These items are assessed as follows:

- **Transition readiness** is assessed by answering the question: “Do you think that you are ready to transfer to adult care?”. Responses range from 1 (“no, definitely not”) to 4 (“yes, definitely”) [32, 33].
- **Global function** is assessed using the PedsQL™ 4.0 Generic Scale, validated for use with 18-25-year-olds [34, 35]. It contains 15 items rated on a 5-point Likert scale (0 = Never, 4 = Almost always). It measures physical, emotional, social, and school function and is an important marker in transition [29, 36].
- **Self-efficacy** is measured using the National Institutes of Health’s Patient-Reported Outcomes Measurement Information System (PROMIS®). PROMIS Self-Efficacy for Managing Chronic Conditions questionnaires asks patients to rate their confidence of performing tasks across six domains: Daily Activities, Symptoms, Medications and Treatments, Emotions, Social Interactions, and Informational Support [37, 38].
- **Disease activity** will be documented using a Physician Global Assessment (PGA). A PGA will be performed and documented by the pediatric rheumatologist at baseline and the adult rheumatologist at their first appointment and again at follow-up for every participant. The PGA is measured on a 21 point Likert scale between 0 to 10 reflecting no disease activity to most active disease [39].

Finally, satisfaction questionnaires will be administered to both the participants and coaches upon completion of the study. This will include quantitative and qualitative review of the intervention including the number of appointments, content covered, and overall impression of the program and suggestions for feedback.

### Data management

Study data are collected and managed using Research Electronic Data Capture (REDCap) electronic data capture tools hosted at McMaster University [40, 41]. REDCap is a secure, web-based software platform designed to support data capture for research studies, providing 1) an intuitive interface for validated data capture; 2) audit trails for tracking data manipulation and export procedures; 3) automated export procedures for seamless data downloads to common statistical packages; and 4) procedures for data integration and interoperability with external sources. The research assistant conducts chart reviews and enters data into REDCap. Questionnaires completed by study participants are directly entered into REDCap upon completion. REDCap is monitored regularly for completion of data collection.

### Safety considerations

There is no change to the participant’s rheumatology care in either group; both will have follow-up assessments by their treating rheumatologists as clinically indicated. There are no changes or restrictions to medication use or any other therapies.

If any mental health concerns are identified in the coaching sessions or on the screening questionnaires, the transition coach will inquire about current treatments and supports. Should further follow up or more support be required, the transition coach will contact the patient’s family physician via their rheumatologist. The social worker transition coach may also connect with a local adolescent medicine specialist for further support if required.

### Data analyses

We will use univariate analyses including frequencies and proportions to report all feasibility outcomes related to the primary aim, in addition to satisfaction questionnaires. Open-ended satisfaction questionnaire results from participants and transition coaches will be used to inform modifications and refinement of the transition coaching intervention for the future multi-centre RCT.

For the secondary aim, descriptive statistics will summarize demographic characteristics and clinical outcomes at each time point. Baseline descriptors will also be summarized by sex and gender. The study is not powered to detect statistical significance between the groups; rather these results will be used to calculate a sample size for a future fully powered study.

Data will be analysed by a member of the research team using SPSS v.26 (IBM, New York, United States). This team member will not have access to any identifying information.

### Ethical considerations and declarations

This study has received approval from our McMaster University REB (#14499) and Western University REB (#121456). This study was registered to ClinicalTrials.gov with protocol ID: 14499, registration number: NCT05545839. Full study protocol as approved by REB is included in S1 Protocol. SPIRIT checklist available in S1 Checklist.

### Status and timeline of the study

The study started recruitment in October 2022 at the primary site and March 2023 at the secondary site. Recruitment is expected to last 19 months, through to Spring/Summer 2024. Following that, data collection will be complete 11 months after the last patient is recruited.

## Discussion

Numerous transition interventions have been studied, primarily related to planning for transition and administrative assistance with the transition process [17, 18]. This commonly includes assistance with preparing transition documentation, transfer of information, and coordinating appointments [18, 42]. Generally, the existing examples have lacked the longitudinal and structured intervention of the TRACER TC program, as well as rigorous design with randomization and a formal control group.

A few prospective, intervention-based programs in pediatric rheumatology have been described but a clear consensus on the benefit has not been established. A social worker-based follow-up program in the United States described by Jensen et al. demonstrated improved follow-up rates [43]. This program however was not randomized, with the control group consisting of those who were eligible but did not undergo initial assessment [43]. Similarly, a program in Belgium employed the use of a transition coordinator, primarily pre-transfer, and followed a group of patients transitioning out of pediatric care. Likewise, the control group was a retrospective cohort [44]. A clinic based in the United States provided with virtual modules with some similar content to the TC sessions which will be completed in TRACER (medication management, plans of care/emergency planning), and enrolled patients at their rheumatology transition clinic. There was no control group, but they did find that most patients participated in at least one module and had improved initial attendance rates at adult care compared to previously reported literature [45]. This was however a standardized curriculum, which did not allow for individualized tailoring and connection, which TRACER will incorporate.

The TRACER protocol has been designed to collect robust and high-quality data around the impact of healthcare coaching on self-efficacy and self-management skills during and immediately following the transition from pediatric to adult care.

The current study is designed as a feasibility study, which, by design, means that the sample size will not be adequate to examine the intervention’s effectiveness. Outcomes from this feasibility trial will inform about recruitment rates that we can anticipate in a future study and the potential impact of the intervention for power calculation. Limitations of this feasibility study include language and accessibility. Due to the limited scale, the transition coaching intervention is only available to those who can communicate verbally in English. Given the multicultural nature of Canada, it would certainly be valuable to broaden the language inclusivity in the larger multi-site study.

This study is currently designed for and recruiting patients with pediatric rheumatic disease, with all patients receiving rheumatologic care eligible for recruitment. This inclusivity will allow for the study to be applicable to typical rheumatology practice but may mask difference between different groups of patients. A similar protocol currently being tested locally for pediatric patients with inflammatory bowel disease as well. If this protocol is demonstrated to be feasible for a multi-site randomized control trial, the transition coaching intervention could be applied more broadly with confidence to other pediatric-onset chronic diseases as well. Results of this feasibility trial will be communicated with the scientific community in the form of a peer reviewed publication and presentation at an annual scientific conference.

## Supporting information

S1 Checklist SPIRIT clinical trial protocol checklist. (DOC)

S1 File Youth transition roadmap; transition to adult rheumatology care. (PDF)

S1 Protocol Complete study protocol as approved by local research ethics board. (DOCX)
