# Supplementary material for: Transition to Adulthood through Coaching and Empowerment in Rheumatology (TRACER): A feasibility study protocol
Source: PLoS One. 2024 Aug 26;19(8):e0295174. doi: 10.1371/journal.pone.0295174 (PMC11346723; doi:10.1371/journal.pone.0295174)
Supplement: S1 File — (PDF) [file pone.0295174.s002.pdf]

Appendix A

YOUTH TRANSITION ROAD MAP

Transition to Adult Rheumatology Care

McMaster Children's Hospital
HAMILTON HEALTH SCIENCES

Transition to Adult Rheumatology Care

"Healthcare transition" describes the gradual process of getting ready to transfer from pediatric to adult care around age 18.

Thinking about transition: The team will talk about the idea of transition and explore your skills, abilities and understanding of how to manage your health.

Planning your transition: With support from your pediatric team, the responsibility for your health care slowly shifts from your parent/caregiver to you.

Making the transition: The time when your care will be shifted from the pediatric team to adult care.

How to use the Road Map

The goal of this road map is to help you make the transition from pediatric to adult rheumatology care. In it, we outline things that are important to know before you make the transition.

At your own pace, work through the road map and try to accomplish some of the tasks on your own.

Some tasks will be easier than others, and that's OK.

Take your time. You don't have to work through it in order. Start with goals that seem manageable and work from there.

You don't have to have every goal achieved before you turn 18. This road map can be taken with you to adult care and you can continue to use it to gain independence.

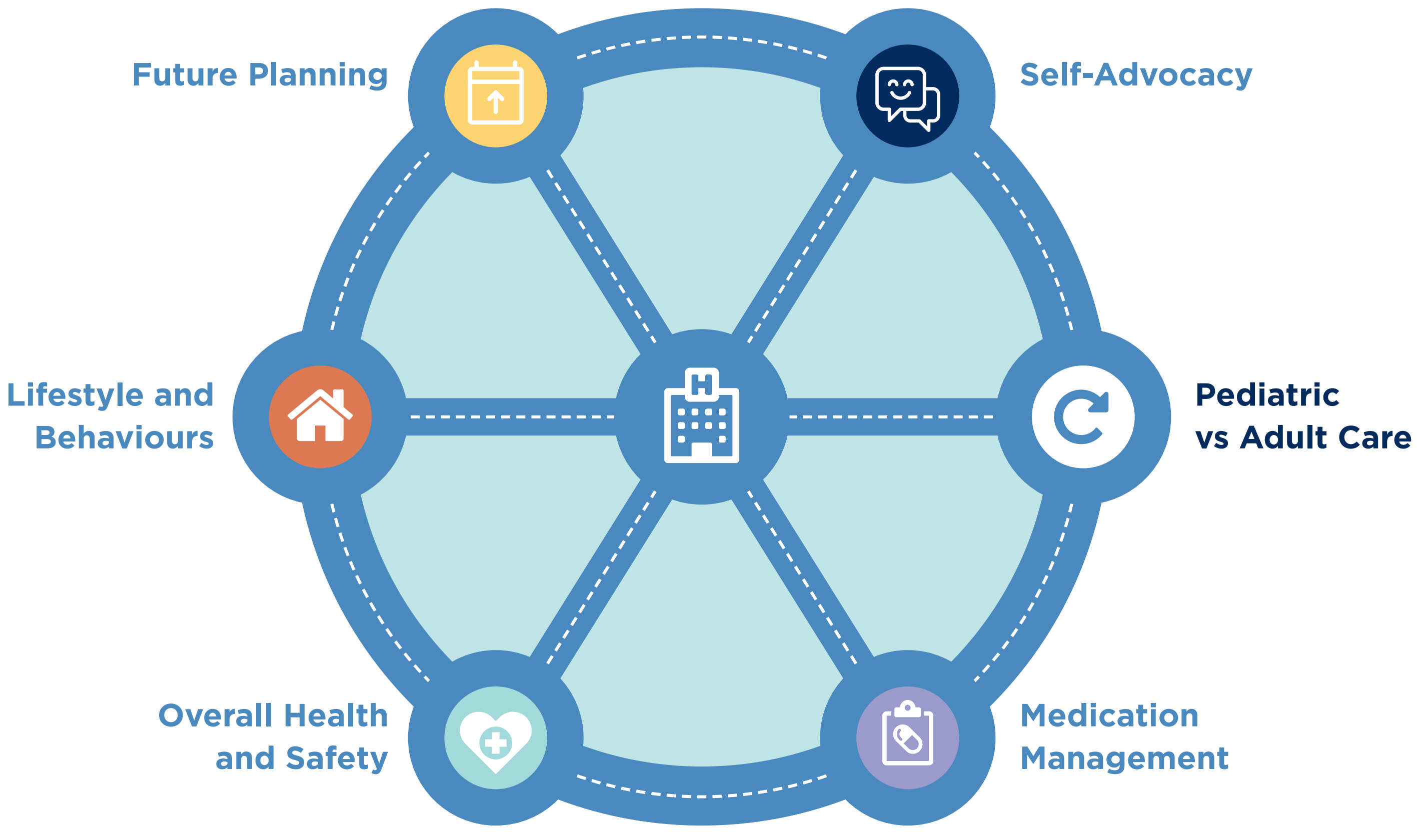

What are the differences between pediatric and adult care?

There are several differences between the care provided in adult and pediatric rheumatology. This table outlines some important differences for you to be aware of.

	Pediatric Care	Adult Care
**Practice Approach**	Family-centered; Parents very involved; shared decision making with parents	Patient-centered; Independence is strongly encouraged; shared decision making with young adult
**Care Provider**	Multi-disciplinary team members are involved with appointments	Clinic visits may include only the doctor and not a team of people
**Clinic Location**	In children's hospital	Private office-based settings or hospital clinic
**Length of Appointment**	Longer time	Shorter time
**Parent/ Caregiver Presence in Clinic**	Parent/caregiver usually present during visit	Patient usually seen alone unless requests and consents to parent/caregivers presence
**Information Sharing**	Information shared with parents/caregivers	Information is shared with only the patient

Health care for youth is different than for adults.

In an adult care setting, you will manage your own care. You can involve your parents/caregivers if you choose. This table describes how your role changes as you move from pediatric to adult care.

In Pediatric Care, your PARENT/CAREGIVER:	Adult Care, YOU:
Is usually with you at your appointments	See the doctor alone and only ask someone to join you if you choose
Answers questions from the doctor and explains how you have been doing and what medicine you're taking	Answer questions from the doctor and explain how you've been doing and what medications you've been taking
Is involved in making decisions about your treatments	Make decisions about your treatments and ask your parent/caregiver only when needed
Helps schedule appointments, lets the office know if you need to change an appointment and picks up your prescriptions	Schedule your own appointments, let the office know if you need to change an appointment and pick up your own prescriptions
Helps you follow a treatment plan and reminds you to take medications	Take responsibility for following a treatment plan and take medications on your own
Has access to your health information, treatments and test results	Have access to your own health information which is confidential unless you allow someone else to see it
Has your health insurance information	Have your own insurance card and understand your coverage
Keeps a record of your medical history and immunizations	Keep a record of your own medical history and immunizations

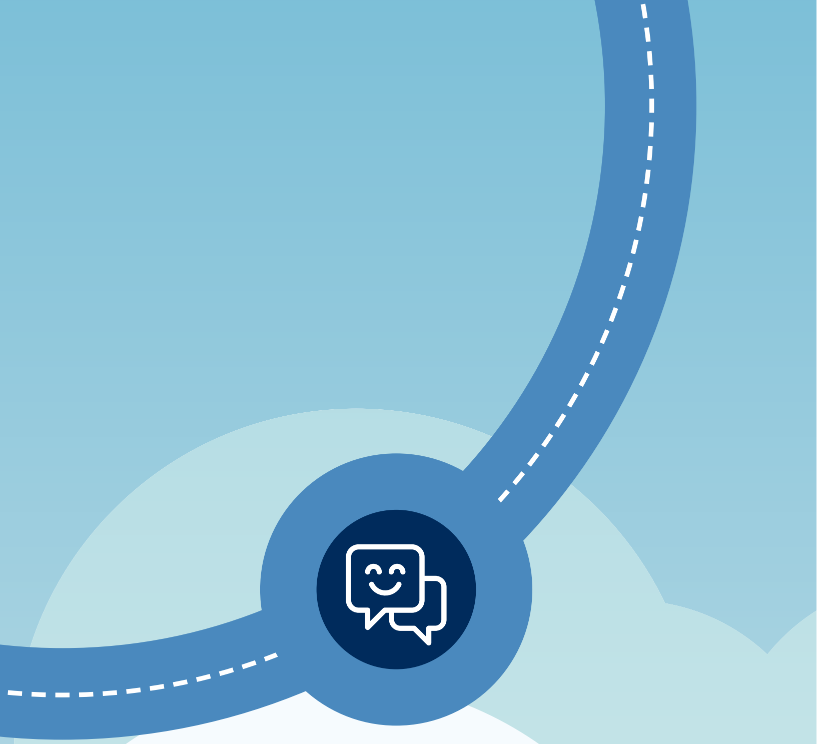

Self-Advocacy

This is a skill where you learn to speak up for yourself and let your doctor (or team) know your concerns and questions. Ask your parents/caregiver and your team if you don't know what some of the terms on this list mean. Start writing down questions and bringing them to your appointment.
This is all **SELF-ADVOCACY!**

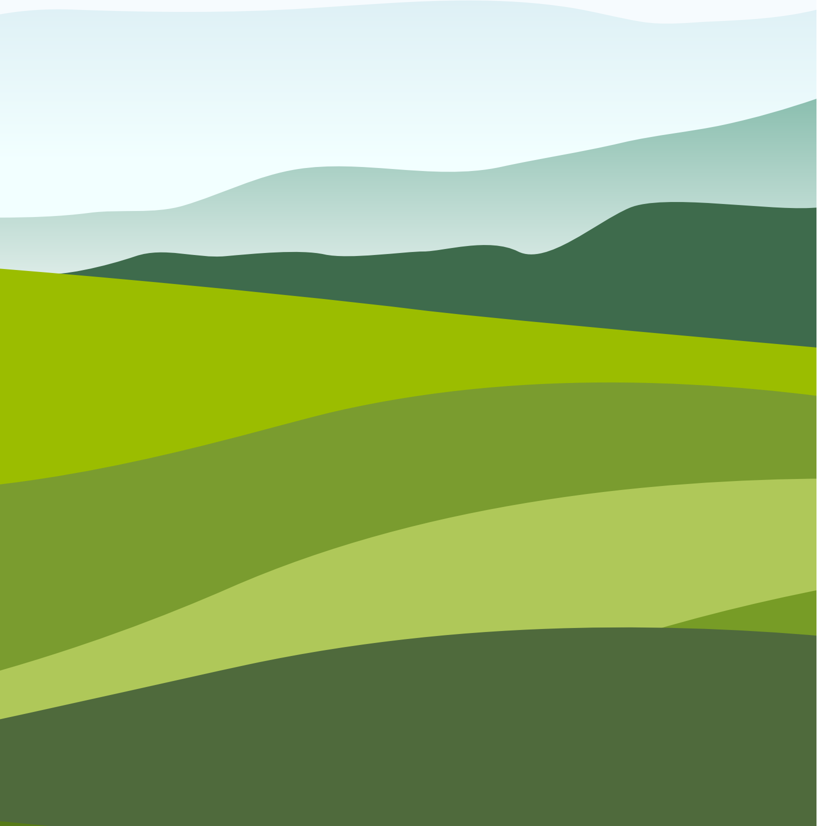

Self-Advocacy

- [ ] I ask my healthcare provider questions about my health at each visit
- [ ] I am comfortable meeting with my healthcare providers on my own
- [ ] I understand what patient privacy and confidentiality means (for example, who can get information about your health or who can know about it)
- [ ] I understand what it means to consent to healthcare treatments
- [ ] I ask for what I need from my healthcare provider
- [ ] I can describe my disease to someone when I am asked
- [ ] I know how/where to access mental health support if I need it
- [ ] I know how to advocate for myself if I require special supports in my school (college or university) or my workplace (special seating, keyboard, access to note taker, accommodation for exams, arranged dismissals, etc.)

Medication Management

Medication Management

There is often a lot of information to learn about your medications, what they are for, and what they can do. Take your time, get to know your medications, and ask your doctor, pharmacist, or nurse if you have any questions.

- [ ] I know the names and doses of the medications I take or have a list of them on me at all times
- [ ] I take my medications without being reminded
- [ ] I know why I take my medications
- [ ] I know the side effects of my medications
- [ ] I refill my own prescriptions before they run out
- [ ] I know how to contact my pharmacist if I need to
- [ ] I know how to store my medications
- [ ] I know how/when to take my medications

General Health

General Health

Not only is it important to be able to care for your health related to rheumatology, it's important to be able to care for your health and safety as a whole. Have your parents/caregiver help you make phone calls, ask them who else is on your healthcare team. You've got this!

- [ ] I carry my own health card
- [ ] I book my own medical appointments
- [ ] I have a family doctor
- [ ] I know all my healthcare providers and who to contact when I need help or have a question
- [ ] I know if I have any medication allergies
- [ ] I know what to do in a medical emergency (who to call, where to go)
- [ ] I will contact my doctor if I need to
- [ ] I understand my tests and procedures (joint injection, surgery, etc.) and any risks associated with them

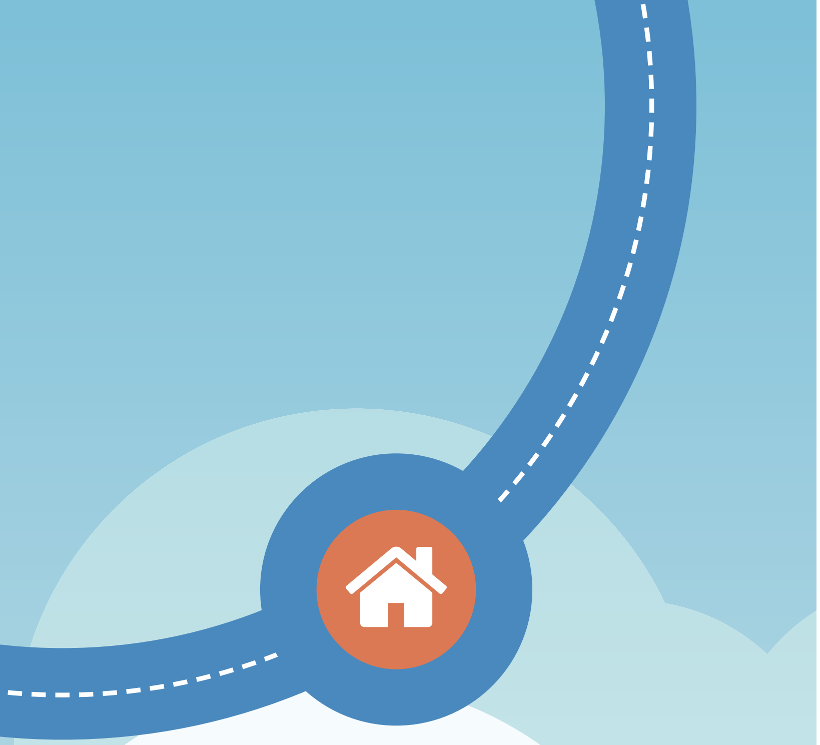

Lifestyle and Behaviours

It's important to have a healthy lifestyle to care for both your overall health and your rheumatologic disease. Ask your team about how alcohol, drugs, smoking, and physical activity can affect your disease. Ask about how your disease and medications may affect pregnancy.

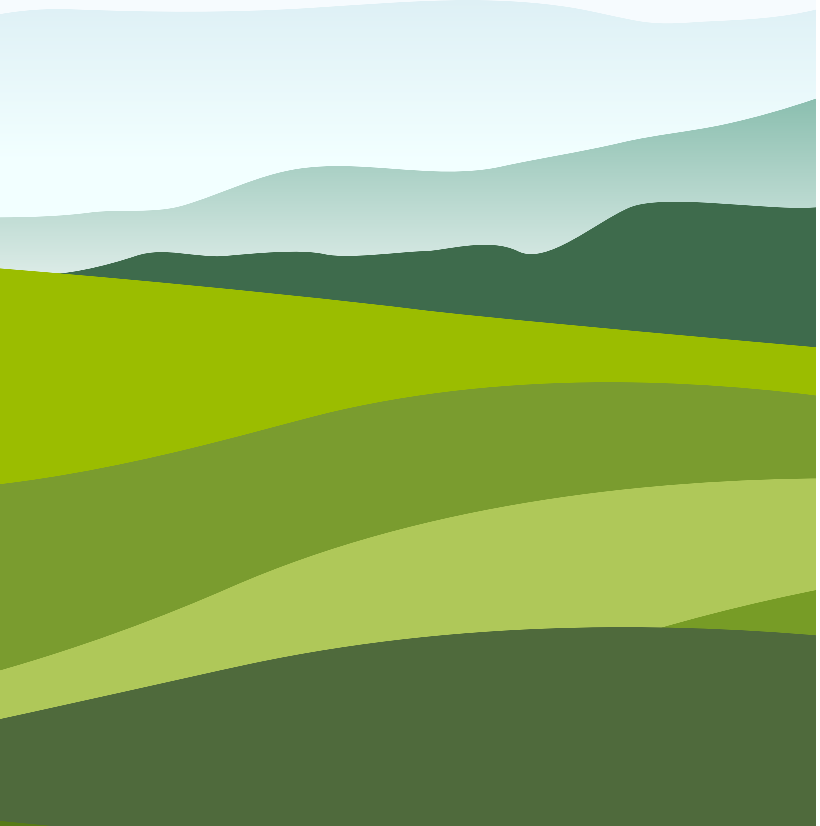

Lifestyle and Behaviours

- [ ] I discussed how alcohol can affect my health and any interaction with my medications
- [ ] I discussed how tobacco, marijuana and other drugs affect my health and any interactions with my medications
- [ ] I understand the importance of a healthy diet and physical activity for my health
- [ ] I know how to prevent unplanned pregnancies and sexually transmitted infections (STIs)
- [ ] I understand how my health and medications could affect pregnancy

Lifestyle and Behaviours

It's important to have a healthy lifestyle to care for both your overall health and your rheumatologic disease. Ask your team about how alcohol, drugs, smoking, and physical activity can affect your disease. Ask about how your disease and medications may affect pregnancy.

Future Planning

- [ ] I discussed my current health/ medication insurance coverage and how that may change over time as I get older
- [ ] I am able to get myself to and from my healthcare appointments
- [ ] I know how to access supports for getting to and from my appointment if I need help
- [ ] I know how to access supports for getting around (being social, visiting, school, shopping, errands)
- [ ] I have an idea about what I want to do in my future (college, university, work) and how I can access resources to help me get there
- [ ] I know how to access financial support for living and school/work expenses if I need it I know how to access support for assistance with driving/getting a driver's license

MyTransition App

The MyTransition App was developed to help prepare adolescents for the transition from pediatric to adult care. The app includes tools to describe one's health condition, keep track of members of their healthcare team, and create a **"MyHealth 3-Sentence Summary"**.

This summary allows you to keep a brief summary of the important aspects of your medical condition in one place. The MyTransition App also includes a way to measure skills related to managing one's own health through the **Transition-Q**.

The **Transition-Q** is a 14-item transition readiness and selfmanagement ability score. You can use this score to set personalized transition goals within the app and track your progress over time.

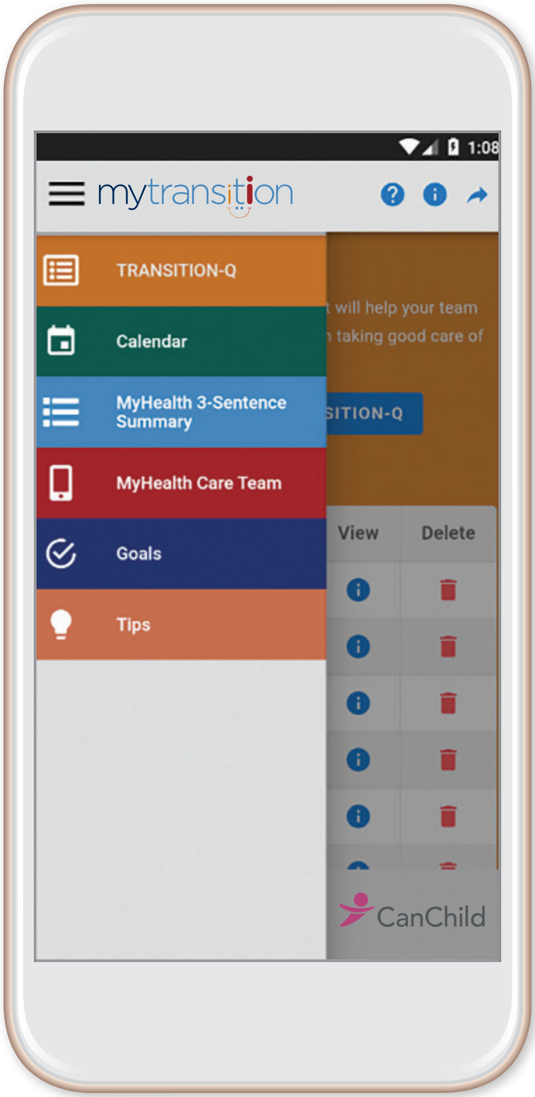

The MyTransition app is available to download for free on iPhone and Android devices.

Scan for MyTransition App:

Apple

Android

Resources and Contact Information:

Information about our projects:

https://canchild.ca/en/research-in-practice/current-studies/trust-study
