# Supplementary material for: Transition to Adulthood through Coaching and Empowerment in Rheumatology (TRACER): A feasibility study protocol
Source: PLoS One. 2024 Aug 26;19(8):e0295174. doi: 10.1371/journal.pone.0295174 (PMC11346723; doi:10.1371/journal.pone.0295174)
Supplement: S1 Protocol — (DOCX) [file pone.0295174.s003.docx]

**Title of Study:** TRACER: Transition to Adulthood through Coaching and Empowerment in Rheumatology, A Feasibility Study

**Locally Responsible Investigator and Principal Investigator, Department/Hospital/Institution:**

Michelle Batthish, MSc, MD, FRCPC

Pediatric Rheumatologist, McMaster Children’s Hospital

**Sponsor**: The Arthritis Society

## 1.0 Background

Health Care Transition (HCT) is a purposeful, planned process when youth progressively assume more responsibility for their health as they prepare to transfer from pediatric to adult care ^1,2^. Importantly, HCT should be informative and provide developmentally appropriate psychosocial support. ^1,2^. A poorly managed transition can have adverse effects on the quality and experience of care as well as contribute to poor disease outcomes including increased morbidity and even mortality ^1,3-7^. In fact, youth frequently report a negative experience during transition and as many as 50% disengage entirely from care ^4^. Despite knowing that the transition period is an extremely vulnerable time for youth with chronic disease, high quality research, evidence-based transition programs and consensus of what defines/determines a successful transition remain elusive ^8,9^.

While undergoing HCT, youth also experience critical life transitions that include changes to their biology (e.g., puberty), social networks (e.g., leaving parental home), and educational/vocational pursuits ^10,11^. Unfortunately, however, support for youth during this period (known as third phase of HCT - age 18-25) is often neglected in clinical care and is disproportionally under-researched ^2,8,12-17^ compared to earlier phases.

Health Coaching (HC) has been defined as “helping patients gain the knowledge, skills, tools and confidence to become active participants in their care so that they can reach their self-identified health goals” ^18^. For patients with early-stage kidney disease, HC demonstrated positive effects on quality of life, self-management, patient activation and self-efficacy after only six weeks of support and maintained its effects twelve weeks post intervention ^19^. While HC has been described as an adjunct form of support in rheumatology care ^20^, there is great potential for this role to collaborate with adult rheumatologists to optimally support youth after they transition to adult care.

The concept of HC was implemented at McMaster’s Young Adult Rheumatology Clinic in March 2020 in response to the COVID-19 pandemic. An ACPAC therapist supporting an adult rheumatologist (Dr. Matsos) adapted service delivery by connecting virtually with each young adult one week prior to their appointment with the adult rheumatologist. This virtual meeting created dedicated time to provide the necessary education and support required in this third stage of HCT. Many stakeholders have noticed positive benefits of this program, including youth feeling more confident in their care, the adult rheumatologist noticing fewer ‘no shows’ and parents expressing relief that their children were well supported. As this positive feedback persisted, these sessions evolved further into individualized Health Coaching Interventions (HCI), guiding the young adults to gain knowledge, skills, tools and confidence while still providing medical care and supporting the young adults psychosocial, educational, and vocational needs during these virtual sessions.

## 2.0 Study Objectives

1. To assess the feasibility of conducting a multi-center, randomized-controlled trial to answer the following research question: To what extent does the implementation of a Youth Transition Roadmap (YTR) and virtual Transition Coach Intervention (TCI) improve self-management skills over the YTR alone in youth being transferred from pediatric to adult rheumatology care?
   1. The follow criteria will be used to assess feasibility: 1) Consent ≥85% of patients approached; 2) enroll ≥30% from non-primary site (London); 3) ≥90% attendance of virtual sessions; 4) complete ≥90% of outcome assessments at 8-month follow-up (TCI complete); and 5) have ≤5% missing data
2. To explore clinical outcomes at baseline, 8- and 11-months to characterize our study population and understand variability in changes over time in self-efficacy that may be used to power a large, multicentre RCT.

# 3.0 Methods

This randomized-controlled feasibility trial will recruit patients over 19-months from 2 centers (Hamilton ON and London ON).

## 3.1 Study population, inclusion and exclusion criteria

All 17- and 18-year-olds with a pediatric-onset rheumatic disease attending their last pediatric rheumatology appointment prior to transferring to adult care will be assessed for eligibility by the pediatric rheumatologist (Drs. Batthish, Cellucci, and Heale at McMaster, Dr. Berard at Western). Participants will be eligible if they are able to communicate in English, have access a device capable of videoconference or a phone and are available over the subsequent 8-months. Those with a cognitive impairment preventing participation in the individualized education sessions as determined by the medical staff will be excluded. As a feasibility trial, we will document the number of patients who are ineligible and the reason for ineligibility to address this in the future study.

## 3.2 Recruitment

Pediatric rheumatologists will identify patients attending their scheduled last pediatric rheumatology appointment before transferring to adult care. The pediatric rheumatologist will inform the patient about the study and document reasons for ineligibility, as applicable. Interested patients will be directed to the a research assistant who will provide further information and obtain consent.

## 3.3 Sample size

Our sample size is based on the more conservative of our two primary feasibility outcomes: ≥90% of TCI sessions attended. Assuming this, we will require a sample size of 97 for estimating the expected proportion with 6% absolute precision and 90% confidence ^21^. With an average of 7 patients transferring from pediatric to adult care each month (5 Hamilton, 2 London), we will approach 133 patients over 19 months. With an 85% consent rate ^22^ and a 90% study completion rate (133 x 85% x 90% = 102), we will ensure that we meet our sample target of 97 participants completing the study.

## 3.4 Randomization

Consenting participants will be randomly allocated to the Transition Coach Intervention (TCI) or the control group using the Robust Randomization App ^23^ to generate a site-stratified computer-generated block randomization list with random varying permutated blocks of 4 and 6 and a 1:1 allocation ratio.

## 3.5 Intervention

All participants (TCI and control group) will receive a paper and electronic copy of the Youth Transition Roadmap (YTR) ^24^, which informs youth about differences between adult and pediatric care and discusses 5 domains of healthcare transition; Self-Advocacy, Medication Management, General Health, Lifestyle Issues and Future Planning related to education and vocation.

All participants will be scheduled for their first adult rheumatology visit (Drs. Haig, Matsos, Garner) within 3-months of consent. The timing of subsequent clinical visits will be at the adult rheumatologist’s discretion. All participants will receive standard of care from their rheumatologists.

Contact information for participants allocated to the TCI group will be provided to the Transition Coaches (TCs) who will schedule participants for their first TC session within 1 month of consent. Sessions with the TCs will be virtual (by videoconference or phone, at the participant’s preference) and will be independent of clinical visits with the adult rheumatologists. There will be 8 monthly sessions, 6 with as ACPAC physiotherapist and 2 with a social worker. The topics will be as follows:

ACPAS physiotherapist transition coach sessions:

- Pediatric to adult care
- Self-advocacy
- Medication management
- General health
- Lifestyle and behaviours
- Future planning

SW transition coach sessions:

- Screening for anxiety and depression (PHQ4)
- Depending on the participant’s self-identified areas of need, topics including:
  - Disease severity, daily impact, current treatment and possible side effects
  - Biologic life transitions
  - Self-management skills
  - Pain management and coping strategies
  - Body image, confidence
  - Living situation, parental/sibling support
  - Ability to partake in social/recreational activities
  - Social relationships/networks
  - Sleep/motivation/energy
  - Anxiety, depression, anger, denial (grief, feelings associated with loss)
  - Lack of validation from others
  - Communicating effectively with medical team/self-advocacy
  - Coping/accommodations with school
  - Being different than others
  - Becoming aware of negative thought patterns and education surrounding learning to recognize depressive thought patterns and strategies
  - Uncertainty/fear about the future

Neither TC will have any contact with the control group for the duration of the study to minimize potential for cross contamination.

If any concerns be identified in the coaching sessions or on the screening questionnaires, the transition coach will inquire about current treatments and supports. Should further follow up/support be required, the transition coach will contact the patient's family doctor via their rheumatologist. The social worker transition coach may also connect with Dr. Grant (adolescent medicine specialist) for further support if required.

Questionnaires evaluating global function, self-efficacy and transition readiness will be administered at baseline, 8 months and 11 months.

# 4.0 Outcomes

This study is a pilot/feasibility study, which will be used to inform a multi-centre trial measuring the effect of transition coaching. The data required for the primary outcome includes rates of consent/recruitment, coaching session completion and data collection.

# Measurements and Measurement Instruments

Demographic data: to be collected on from chart review (by research assistant) at enrollment:

- 1. Age
  2. Sex (male, female, intersex)
  3. Gender (self-report; man, woman, non-binary, prefer not to say)
  4. Disease type
  5. Current medications
  6. Age at diagnosis
  7. Family history of rheumatic disease
  8. Comorbidities
  9. Vocation (employed/college/university/other)
  10. Estimate of household income (using postal code)
  11. Newest Vital Sign© (measure of health literacy) ^25^.

At baseline, 8-months (completion of TCI) and 11-months (3-months following TCI completion), the following will be collected:

1. Transition readiness: We will measure attitude towards transition by asking participants to what extent they agree with each of 4 statements on a five-point Likert scale ^9,26^. Readiness for transition will be assessed by asking a single question: “Do you think that you are ready to transfer to adult care?”. Responses range from 1 (“no, definitely not”) to 4 (“yes, definitely”) ^26,27^.
2. Global functional assessment: PedsQL™ 4.0 Generic Scale, validated for use in 18-25 year-olds ^28,29^, contains 15-items rated on a 5-point Likert scale (0=Never, 4=Almost always). It measures physical, emotional, social, and school functioning and is considered one of the most important measures of transition ^22,30^.
3. Disease activity: The active joint count and Physician Global Assessment (PGA) will be performed by the pediatric rheumatologist at baseline and the adult rheumatologist at their first appointment and again at follow-up for every participant. Adult rheumatologists will be blinded to assessments of the pediatric rheumatologist. The PGA is measured on a scale of 0 to 10 reflecting no disease activity to most active disease ^31^.
4. Self-efficacy: We will use the National Institutes of Health’s Patient-Reported Outcomes Measurement Information System (PROMIS®). The PROMIS® Self-Efficacy outcomes for Chronic Disease ^32,33^ evaluate the confidence of patients in performing important tasks and behaviors for managing chronic diseases, and measure one’s belief about their ability to perform behaviors to reach their health goals related to physical function, mental health and social interactions: Self-Efficacy for Managing 1) Daily Activities; 2) Emotions; 3) Managing Medications and Treatments; 4) Social Interactions; 5) Symptoms, and 6) Informational Support ^32^.

|  | Baseline | Month | | | | | | | | | | |
| --- | --- | --- | --- | --- | --- | --- | --- | --- | --- | --- | --- | --- |
|  |  | 1 | 2 | 3 | 4 | 5 | 6 | 7 | 8 | 9 | 10 | 11 |
| Recruitment | X |  |  |  |  |  |  |  |  |  |  |  |
| Demographics | X |  |  |  |  |  |  |  |  |  |  |  |
| Transition readiness | X |  |  |  |  |  |  |  | X |  |  | X |
| Global function assessment | X |  |  |  |  |  |  |  | X |  |  | X |
| Disease activity | X |  |  | X* |  |  |  |  |  |  |  | X* |
| Self-efficacy (PROMIS) | X |  |  |  |  |  |  |  | X |  |  | X |
| Physio TC Intervention |  | X† | X† | X† | X† | X† | X† |  |  |  |  |  |
| SW TC Intervention |  |  |  |  |  |  |  | X† | X† |  |  |  |
| Participant satisfaction |  |  |  |  |  |  |  |  |  |  |  | X |

* First adult rheumatologist visit will occur within 3 months of consent (last scheduled pediatric visit). Follow-up after that will be as clinically indicated. Follow-up disease activity will thus be recorded when the patients return to assessment within the 11-month period

† The order of the physiotherapist and social worker transition coach sessions is not prescribed, but the sessions will occur once a month for 8 months, for the intervention group.

Additional: coach satisfaction surveys, to be completed at the end of the study

# 5.0 Statistical Analysis

We will use frequencies (numbers and proportions) to report all feasibility outcomes related to Aim 1 including satisfaction questionnaires. Open-ended responses from participants and TCs will be used to inform modifications and refinement of the TCI for the fully powered study.

For Aim 2, descriptive statistics will summarize demographic characteristics and clinical outcomes at each time point. Baseline descriptors will also be summarized by sex and gender.

We will examine within-person, within-group and between-group changes in each PROMIS® outcome and will perform sub-analyses by sex and gender. We will determine means, medians, standard deviations and interquartile ranges as appropriate, at baseline and both follow-up times and calculate variability in changes over time. We will not perform statistical analyses of these changes or group differences as we are underpowered to detect them but will use these to calculate a sample size for a future fully powered study.

Data will be analysed using SPSS v.26 (IBM, New York, United States). Data analyses will be supervised by Drs. Ioannidis and Beattie who will support trainees in developing data analysis skills.

# 6.0 Assurance of Protocol Adherence

Completed questionnaires will be reviewed for accuracy and completeness by the research co-ordinator at the time of completion. Protocol deviations will be reviewed to correct any problems.

# 7.0 Feasibility

This primary objective of this study is to investigate the feasibility of studying this intervention and scaling it to multiple centres. A similar concept has been implemented successfully at McMaster’s Young Adult Rheumatology Clinic in March 2020 in response to the COVID-19 pandemic. An ACPAC therapist supporting an adult rheumatologist (Dr. Matsos) adapted service delivery by connecting virtually with each young adult one week prior to their appointment with the adult rheumatologist. This virtual meeting created dedicated time to provide the necessary education and support required in this third stage of HCT. As this positive feedback persisted, these sessions evolved further into individualized Health Coaching Interventions (HCI), guiding the young adults to gain knowledge, skills, tools and confidence while still providing medical care and supporting the young adults psychosocial, educational, and vocational needs during these virtual sessions.

All the pediatric rheumatology staff at McMaster are investigators on this project which will allow for comprehensive recruitment of patients transitioning from pediatric to adult care. This study also is being completed in the context of previous work done by the group investigating transitional care and establishing an environment conducive to high quality transition practices through the support of the pediatric and adult rheumatology teams, a leading health care transition research team and a growing number of patient advocates interested in this initiative.

# 8.0 Generalizability

McMaster Children’s Hospital and LHSC’s Children’s hospitals are pediatric hospitals that provides care to approximately 900,000 patients ≤19 years of age in Southern/Southwestern Ontario ^34,35^. This includes both urban and rural regions, sizeable Aboriginal and immigrant communities and a broad socioeconomic demographic. As a population representative of the overall Canadian population, our results will be widely generalizable.

# References

1. Lawson EF, Mellins ED. Paediatric rheumatic diseases: Navigating the transition from paediatric to adult care. *Nat Rev Rheumatol*. Mar 2017;13(3):138-139. doi:10.1038/nrrheum.2017.16

2. Willis E MJ. Shifting the mindset—adolescent and young adult rheumatology in transition. *Lancet Rheumatol*. 2020;2(4): E236-E244.

3. Campbell F, Biggs K, Aldiss SK, et al. Transition of care for adolescents from paediatric services to adult health services. *Cochrane Database Syst Rev*. Apr 29 2016;4:CD009794. doi:10.1002/14651858.CD009794.pub2

4. Hazel E, Zhang X, Duffy CM, Campillo S. High rates of unsuccessful transfer to adult care among young adults with juvenile idiopathic arthritis. *Pediatr Rheumatol Online J*. Jan 11 2010;8:2. doi:10.1186/1546-0096-8-2

5. Felsenstein S, Reiff AO, Ramanathan A. Transition of Care and Health-Related Outcomes in Pediatric-Onset Systemic Lupus Erythematosus. *Arthritis Care Res (Hoboken)*. Nov 2015;67(11):1521-8. doi:10.1002/acr.22611

6. Oen K. Long-term outcomes and predictors of outcomes for patients with juvenile idiopathic arthritis. *Best Pract Res Clin Rheumatol*. Jul 2002;16(3):347-60.

7. Hersh AO, Pang S, Curran ML, Milojevic DS, von Scheven E. The challenges of transferring chronic illness patients to adult care: reflections from pediatric and adult rheumatology at a US academic center. *Pediatr Rheumatol Online J*. Jun 8 2009;7:13. doi:10.1186/1546-0096-7-13

8. McDonagh JE, Farre A. Transitional Care in Rheumatology: a Review of the Literature from the Past 5 Years. *Curr Rheumatol Rep*. Sep 6 2019;21(10):57. doi:10.1007/s11926-019-0855-4

9. Stinson J, Kohut SA, Spiegel L, et al. A systematic review of transition readiness and transfer satisfaction measures for adolescents with chronic illness. *Int J Adolesc Med Health*. 2014;26(2):159-74. doi:10.1515/ijamh-2013-0512

10. Palman J, McDonagh JE. Young Minds: Mental Health and Transitional Care in Adolescent and Young Adult Rheumatology. *Open Access Rheumatol*. 2020;12:309-321. doi:10.2147/OARRR.S228083

11. Petty R. Chapter 12: Moving From Pediatric to Adult Rheumatology Care. *Pediatric Rheumatology*. 2017:133-8.

12. Hart LC, Patel-Nguyen SV, Merkley MG, Jonas DE. An Evidence Map for Interventions Addressing Transition from Pediatric to Adult Care: A Systematic Review of Systematic Reviews. *J Pediatr Nurs*. Sep - Oct 2019;48:18-34. doi:10.1016/j.pedn.2019.05.015

13. Foster HE, Minden K, Clemente D, et al. EULAR/PReS standards and recommendations for the transitional care of young people with juvenile-onset rheumatic diseases. *Ann Rheum Dis*. Apr 2017;76(4):639-646. doi:10.1136/annrheumdis-2016-210112

14. Selvaag AM, Aulie HA, Lilleby V, Flato B. Disease progression into adulthood and predictors of long-term active disease in juvenile idiopathic arthritis. *Ann Rheum Dis*. Jan 2016;75(1):190-5. doi:10.1136/annrheumdis-2014-206034

15. Groot N, Shaikhani D, Teng YKO, et al. Long-Term Clinical Outcomes in a Cohort of Adults With Childhood-Onset Systemic Lupus Erythematosus. *Arthritis & rheumatology*. Feb 2019;71(2):290-301. doi:10.1002/art.40697

16. Nordal E, Zak M, Aalto K, et al. Ongoing disease activity and changing categories in a long-term nordic cohort study of juvenile idiopathic arthritis. *Arthritis Rheum*. Sep 2011;63(9):2809-18. doi:10.1002/art.30426

17. Samarasinghe S MS, Ho J, Steinbeck K. Chronic illness and transition from paediatric to adult care: a systematic review of illness specific clinical guidelines for transition in chronic illnesses that require specialist to specialist transfer. *J Trans Med*. 2020;<https://doi.org/10.1515/jtm-2020-0001>

18. Bennett HD, Coleman EA, Parry C, Bodenheimer T, Chen EH. Health coaching for patients with chronic illness. *Fam Pract Manag*. Sep-Oct 2010;17(5):24-9.

19. Lin MY, Cheng SF, Hou WH, Lin PC, Chen CM, Tsai PS. Mechanisms and Effects of Health Coaching in Patients With Early-Stage Chronic Kidney Disease: A Randomized Controlled Trial. *J Nurs Scholarsh*. Mar 2021;53(2):154-160. doi:10.1111/jnu.12623

20. Kataria S, Ravindran V. Digital health: a new dimension in rheumatology patient care. *Rheumatol Int*. Nov 2018;38(11):1949-1957. doi:10.1007/s00296-018-4037-x

21. Dhand NK KM. Statulator: An online statistical calculator. Sample Size Calculator for Estimating a Single Proportion.

22. McColl J, Semalulu T, Beattie KA, et al. Transition Readiness in Adolescents With Juvenile Idiopathic Arthritis and Childhood-Onset Systemic Lupus Erythematosus. *ACR Open Rheumatol*. Mar 12 2021;doi:10.1002/acr2.11237

23. Tu C, Benn EKT. RRApp, a robust randomization app, for clinical and translational research. *J Clin Transl Sci*. Dec 2017;1(6):323-327. doi:10.1017/cts.2017.310

24. Dushnicky MJ, Beattie, K.A., Batthish, M. . Youth Transition Road Map: Transition to Adult Rheumatology Care at McMaster Children's Hospital. .

25. Mansfield ED, Wahba R, Gillis DE, Weiss BD, L'Abbe M. Canadian adaptation of the Newest Vital Sign(c), a health literacy assessment tool. *Public Health Nutr*. Aug 2018;21(11):2038-2045. doi:10.1017/S1368980018000253

26. van Staa AL, Jedeloo S, van Meeteren J, Latour JM. Crossing the transition chasm: experiences and recommendations for improving transitional care of young adults, parents and providers. *Child Care Health Dev*. Nov 2011;37(6):821-32. doi:10.1111/j.1365-2214.2011.01261.x

27. van Staa A, van der Stege HA, Jedeloo S, Moll HA, Hilberink SR. Readiness to transfer to adult care of adolescents with chronic conditions: exploration of associated factors. *J Adolesc Health*. Mar 2011;48(3):295-302. doi:10.1016/j.jadohealth.2010.07.009

28. Varni JW, Limbers CA. The PedsQL 4.0 Generic Core Scales Young Adult Version: feasibility, reliability and validity in a university student population. *J Health Psychol*. May 2009;14(4):611-22. doi:10.1177/1359105309103580

29. Varni JW, Seid M, Smith Knight T, Burwinkle T, Brown J, Szer IS. The PedsQL in pediatric rheumatology: reliability, validity, and responsiveness of the Pediatric Quality of Life Inventory Generic Core Scales and Rheumatology Module. *Arthritis Rheum*. Mar 2002;46(3):714-25.

30. Sabbagh S, Ronis T, White PH. Pediatric rheumatology: addressing the transition to adult-orientated health care. *Open Access Rheumatol*. 2018;10:83-95. doi:10.2147/OARRR.S138370

31. Ringold S, Wallace CA. Measuring clinical response and remission in juvenile idiopathic arthritis. *Curr Opin Rheumatol*. Sep 2007;19(5):471-6. doi:10.1097/BOR.0b013e32825a6a68

32. Cella D, Riley W, Stone A, et al. The Patient-Reported Outcomes Measurement Information System (PROMIS) developed and tested its first wave of adult self-reported health outcome item banks: 2005-2008. *J Clin Epidemiol*. Nov 2010;63(11):1179-94. doi:10.1016/j.jclinepi.2010.04.011

33. Gruber-Baldini AL, Velozo C, Romero S, Shulman LM. Validation of the PROMIS((R)) measures of self-efficacy for managing chronic conditions. *Qual Life Res*. Jul 2017;26(7):1915-1924. doi:10.1007/s11136-017-1527-3

34. Wellington) LHINLHNHBLW. <http://www.hnhblhin.on.ca/aboutus/geographyanddemographics.aspx>; <http://www.waterloowellingtonlhin.on.ca/aboutus/population_snapshot.aspx>.

35. Welcome to the Children’s Hospital! <https://www.lhsc.on.ca/childrens-hospital/welcome-to-the-childrens-hospital>

# Appendix A – PedQL 4.0 Generic Scales (Young Adult version)

Each question is answered on the following scale: Never a problem (0), almost never a problem (1), sometimes a problem (2), often a problem (3), almost always a problem (4)

| **Physical functioning scale** | |
| --- | --- |
| It is hard for me to walk more than one block | |
| It is hard for me to run | |
| It is hard for me to do sport activity or exercise | |
| It is hard for me to lift something heavy | |
| It is hard for me to take a bath or shower by myself | |
| It is hard for me to do chores around the house | |
| I hurt or ache | |
| I have low energy | |
|  |  |
| **Emotional functioning scale** | |
| I feel afraid or scared | |
| I feel sad or blue | |
| I feel angry |  |
| I have trouble sleeping | |
| I worry about what will happen to me | |
|  |  |
| **Social functioning scale** | |
| I have trouble getting along with other young adults | |
| Other young adults do not want to be my friend | |
| Other young adults tease me | |
| I cannot do things that others my age can do | |
| It is hard to keep up with my peers | |
|  |  |
| **Work/school functioning scale** | |
| It is hard to pay attention at work or school | |
| I forget things | |
| I have trouble keeping up with my work or studies | |
| I miss work or school because of not feeling well | |
| I miss work or school to go to the doctor or hospital | |

# Appendix B - Self-efficacy questionnaire (PROMIS)

The following questions are answered on the scale: I am not at all confident (1), I am a little confident (2), I am somewhat confident (3), I am quite confident (4), I am very confident (5)

| **Managing emotions** |
| --- |
| I can handle negative feelings |
| I can find ways to manage stress |
| I can handle upsetting situations |
| I can avoid feeling discouraged |
| I can keep emotional distress from interfering with things I want to do |
| I can bounce back from disappointment |
| I can relax my body to reduce my anxiety |
| I can handle the stress of going for treatment of my medical conditions |
|  |
| **Managing medications and treatment** |
| I can follow directions when my doctor changes my medication |
| I can take my medication when I am working or away from home |
| I can take my medication when there is a change in my usual day |
| I can manage my medications without help |
| I can remember to take my medication as prescribed |
| I can use technology to help me manage my medication and treatments |
| I can list my medications, including the doses and schedule |
| I can figure out what treatment I need when my symptoms change |
|  |
| **Managing social interactions** |
| I can talk about my health problems with someone |
| If I need help, I can find someone to take me to the doctor's office |
| I can get emotional support when I need it |
| I can ask for help when I don't understand something |
| I have someone who helps me understand medical information |
| If I need help, I have someone to help me manage my daily activities |
| I have someone to help me plan and make decisions related to my illness |
| I can communicate well with my doctors and nurses |
|  |
| **Managing symptoms** |
| I can manage my symptoms during my daily activities |
| I can manage my symptoms in a public place |
| I can work with my doctor to manage my symptoms |
| I can keep my symptoms from interfering with my personal care |
| I can manage my symptoms when I am at home |
| I can keep my symptoms from interfering with the work I need to do |
| I can find the information I need to manage my symptoms |
|  |
| **Managing daily activities** |
| I can perform my household chores |
| I can go shopping and run errands |
| I can walk around inside my house |
| I can lift and carry groceries |
| I can take care of others |
| I can manage my clothes when I need to use to the toilet |
| I can keep doing my usual activities at work |
| I can maintain a regularly exercise program |

These last questions are answered on the following scale: Never (1), rarely (2), sometimes (3), usually (4), always (5)

| **Informational support** |
| --- |
| I have someone to give me good advice about a crisis if I need it |
| I have someone to give me information if I need it |
| I get useful advice about important things in life |
| I can get helpful advice from other when dealing with a problem |
| My friends have useful information to help me with my problems |
| I have people I can turn to for help with my problems |
| Other people help me get information when I have a problem |

# APPENDIX C - Intervention Tracking Sheet

|  |  | | 1 | | 2 | 3 | | 4 | | | 5 | 6 | |
| --- | --- | --- | --- | --- | --- | --- | --- | --- | --- | --- | --- | --- | --- |
| **Pediatric to Adult Care** |  | |  | |  |  | |  | | |  |  | |
| See Doctor Alone |  | |  | |  |  | |  | | |  |  | |
| Answer questions |  | |  | |  |  | |  | | |  |  | |
| Make decisions on your own |  | |  | |  |  | |  | | |  |  | |
| Schedule your own appointments |  | |  | |  |  | |  | | |  |  | |
| Take responsibility for medications |  | |  | |  |  | |  | | |  |  | |
| Have control over health information |  | |  | |  |  | |  | | |  |  | |
| Carry health and insurance card |  | |  | |  |  | |  | | |  |  | |
| Keep own health record and immunizations |  | |  | |  |  | |  | | |  |  | |
|  |  | |  | |  |  | |  | | |  |  | |
| **Self-Advocacy** |  | |  | |  |  | |  | | |  |  | |
| Ask Questions |  | |  | |  |  | |  | | |  |  | |
| Meet with all healthcare providers alone |  | |  | |  |  | |  | | |  |  | |
| Understand privacy and confidentiality |  | |  | |  |  | |  | | |  |  | |
| Understand consent |  | |  | |  |  | |  | | |  |  | |
| Ask for what is needed |  | |  | |  |  | |  | | |  |  | |
| Able to describe disease |  | |  | |  |  | |  | | |  |  | |
| Know how to access mental health |  | |  | |  |  | |  | | |  |  | |
| Know how to advocate at school or work |  | |  | |  |  | |  | | |  |  | |
|  |  | |  | |  |  | |  | | |  |  | |
| **Medication Management** |  | |  | |  |  | |  | | |  |  | |
| Know names and doses of medications |  | |  | |  |  | |  | | |  |  | |
| Compliant with medications |  | |  | |  |  | |  | | |  |  | |
| Understand what medications are for |  | |  | |  |  | |  | | |  |  | |
| Know the side effects of medications |  | |  | |  |  | |  | | |  |  | |
| Refill medications on my own |  | |  | |  |  | |  | | |  |  | |
| Know how to contact pharmacist |  | |  | |  |  | |  | | |  |  | |
| Know how to store my medications |  | |  | |  |  | |  | | |  |  | |
| Know how to take my medications |  | |  | |  |  | |  | | |  |  | |
|  |  | |  | |  |  | |  | | |  |  | |
| **General Health** | |  | |  | | |  | |  |  | | |  |
| Carry own health card | |  | |  | | |  | |  |  | | |  |
| Book own medical appointments | |  | |  | | |  | |  |  | | |  |
| Have a family doctor | |  | |  | | |  | |  |  | | |  |
| Know all healthcare providers and how to contact | |  | |  | | |  | |  |  | | |  |
| Know my allergies | |  | |  | | |  | |  |  | | |  |
| Have a plan for medical emergency | |  | |  | | |  | |  |  | | |  |
| Contact the doctor when needed | |  | |  | | |  | |  |  | | |  |
| Understand tests, procedures and risks | |  | |  | | |  | |  |  | | |  |
|  | |  | |  | | |  | |  |  | | |  |
| **Lifestyle and Behaviours** | |  | |  | | |  | |  |  | | |  |
| Alcohol interactions with medications | |  | |  | | |  | |  |  | | |  |
| Tobacco, marijuana and drugs and medications | |  | |  | | |  | |  |  | | |  |
| Healthy eating | |  | |  | | |  | |  |  | | |  |
| Exercise | |  | |  | | |  | |  |  | | |  |
| Sleep | |  | |  | | |  | |  |  | | |  |
| Prevention or pregnancy and STD's | |  | |  | | |  | |  |  | | |  |
| Understanding pregnancy and medications/disease | |  | |  | | |  | |  |  | | |  |
|  | |  | |  | | |  | |  |  | | |  |
| **Future Planning** | |  | |  | | |  | |  |  | | |  |
| Understanding health insurance | |  | |  | | |  | |  |  | | |  |
| Traveling to medical appointments - Driver's License | |  | |  | | |  | |  |  | | |  |
| Access supports for travel if needed | |  | |  | | |  | |  |  | | |  |
| Social circles | |  | |  | | |  | |  |  | | |  |
| Relationships | |  | |  | | |  | |  |  | | |  |
| Hobbies/interests | |  | |  | | |  | |  |  | | |  |
| Education - planning, accessibility | |  | |  | | |  | |  |  | | |  |
| Vocation - planning, accessibility | |  | |  | | |  | |  |  | | |  |
| Financial planning | |  | |  | | |  | |  |  | | |  |

# Appendix D – Participant satisfaction survey

Each question will be answered on the following scale: Strong disagree, disagree, somewhat disagree, neither disagree nor agree, somewhat agree, agree, strongly agree, and have room for free text comments.

| The number of appointments was just right |
| --- |
| The length of time for each appointment was just right |
| The overall time spent (8 months) was just right |
| Topics just right - self-advocacy |
| Topics just right - medication management |
| Topics just right - general health |
| Topics just right - lifestyle and behaviour |
| Topics just right - future planning |
| Topics just right - social work topics |
| Overall, I was satisfied with the Transition Coach Intervention |
| Any other comments, any you would change? |

# Appendix E – Transition coach satisfaction survey

Each question will be answered on the following scale: Strong disagree, disagree, somewhat disagree, neither disagree nor agree, somewhat agree, agree, strongly agree, and have room for free text comments.

| The number of appointments was just right |
| --- |
| The length of time for each appointment was just right |
| The overall time spent (8 months) was just right |
| Topics just right - self-advocacy |
| Topics just right - medication management |
| Topics just right - general health |
| Topics just right - lifestyle and behaviour |
| Topics just right - future planning |
| Topics just right - social work topics |
| Overall, I was satisfied with the Transition Coach Intervention |
| On average, how much additional time did you send on each participant outside of the virtual sessions (in hours) |
| Any other comments, any you would change? |

# Appendix F - Transition Coach Resources for Young Adults

## General Young Adult/Youth Transition Resources

Cassie+ & Friends Resources

<https://cassieandfriends.ca/event/teentransition/>

National Ankylosing Spondylitis Society (NASS) Young Adults

[Online Platform Bringing Young People With AS Together | ASone (nass.co.uk)](http://asone.nass.co.uk/)

Canadian Spondylitis Association young Adult Resources

[Canadian Spondylitis Association | Young Adult Resources](https://spondylitis.ca/young-adult-resources/)

## Alcohol/Drugs

Guideline for youth on medication to learn how it interacts with recreational drugs and alcohol <https://drugcocktails.ca/>

<https://www.camh.ca/-/media/files/canadas-low-risk-guidelines-pdf.pdf>

[https://www.camh.ca/-/media/files/pdfs---reports-and-books---research/canadas-lower-riskguidelines-cannabis-pdf.pdf](https://www.camh.ca/-/media/files/pdfs---reports-and-books---research/canadas-lower-risk-guidelines-cannabis-pdf.pdf)

## Medications

<http://www.drugcocktails.ca/>

## Relationships

NASS Relationships

[Guide to Relationships and Sex with Ankylosing Spondylitis | ASone (nass.co.uk)](http://asone.nass.co.uk/life/relationships-and-sex/)

CSA Guide to Relationships

[Canadian Spondylitis Association | Sex and Spondyloarthritis](https://spondylitis.ca/young-adult-resources/sex-and-spondyloarthritis/)

## Gender

[Gender Resources (bcchildrens.ca)](http://www.bcchildrens.ca/health-info/coping-support/gender-resources)

Mental health

<https://good2talk.ca/>

## Work

Working with a Rheumatic Disease – An interactive tool for youth and young adults [Working with a rheumatic disease (iwh.on.ca)](https://www.iwh.on.ca/archive/working-with-rheumatic-disease/index.html)

Arthritis Society Work and Arthritis

[Arthritis and Work | Arthritis Society](https://arthritis.ca/support-education/arthritis-and-work)

## Post-Secondary Accommodations

Transition Resource Guide for Student with Disabilities – Links to every Ontario University and College Accessibility Office

[Accessibility Services | Transition Resource Guide for Students with Disabilities](https://www.transitionresourceguide.ca/resources/accessibility-services)

## Scholarship opportunities

[Lupus Canada Scholarship | Lupus Canada](https://lupuscanada.org/news/lupus-canada-scholarship/)

[About | UCBeyond Scholarship Program](https://www.ucbeyond.ca/en/about)
